# Manufacturing Instruments

**Published:** 1870-01

**Authors:** 


### ARTICLE V.

### *Manufacturing Instruments.*

By a Student.

In manufacturing excavators, I first obtain the very best material that can be procured at the depots, (Blake & Co). I then forge one end to the size desired, taking care to cause no flaws in the steel, which is quite easily avoided by striking it alternately, that is on one side then on the other, or, which is much better, draw one side out at a time, and by so applying heat each time I can avoid having those flaws, which cause the steel to be worthless. After I have the steel very nearly the size desired, I use a file, which removes all indentations of the hammer, first a course and then a fine file, and so on until smooth. However, after you once become an expert in forging, you will not need a file unless you desire the part forged to be round. Now, being ready to shape according to the pattern desired, I am very careful not to bend while the steel is cold, as it may be tempered from forging to that point which will cause it to be brittle and break. Although when annealed it can be bent to any degree you may wish it is always best to be on the safe side; heat to a *dark red* each time you bend it. Being now ready to temper, I apply such a degree of heat to the end of the instrument as will cause it to become a *cherry red*, and then plunge it into a hard ball of wax. I can not say what temper it is, but this I know, the edge or point is of sufficient hardness to retain a good edge, so as to cleave down the hard tissues of the teeth, and the shank of sufficient hardness to preserve its shape when pressure is applied. Yet if I wish to bend so as to facilitate working or excavating a cavity, I can without fear of breaking, and also return it to the former shape. To sharpen an excavator use an oil stone, or, which is better, an Arkansas stone; after which I polish, by first removing all marks of the file with different grades of emory sticks. These can not be procured at the depots but can be made out of emory paper. I also have another stick, say 8 or 10 inches long by 1 wide, with leather glued upon two of the sides, one side of which contains *crocus*, the other powdered emory with a little sweet oil, using the emory side first, until I have as smooth a surface as can be obtained, afterwards the *crocus* which gives a beautiful polish; or, instead of using the *crocus*, use a burnisher, which will also give a high polish.

The pluggers are shaped in the same manner as the excavators, after which they are ready to serrate, the thickness depending upon the number of serrations desired, and the end being smooth so that the serrations are all of one length, using a file intended for this purpose (serrating). I rub the end of the instrument upon this file, holding it at the same time a little oblique, which is perpendicular with the rows of teeth on the file, until I have small teeth cut upon the end. The direction rubbed must be the same as the rows or teeth on the file; these teeth are never made perfect by this process but must be sharpened, this is done by a small and fine triangular file. This file may be made to have a very fine angle by grinding the one side off, which will enable you to pass between and file the finest serrations. Such work as this requires excellent eyes, but a small hand microscope will facilitate the operation very much. These teeth are required to be sharp and smooth so as not to contain any rough and irregular edges, as the gold will hang to them and prevent you from working with any facility. To make these serrations smooth I use a triangular slip of Arkansas stone, which I sharpen whenever it becomes dull and blunt, so as to pass between these teeth and make each serration smooth and perfect, which is seen by passing the serrations through writing paper. If the perforations made in the paper be perfect, it will not adhere to the instrument when withdrawn, but if imperfect it will adhere and have fine shreds of paper all about the perforations. After these serrations are perfect the instrument is ready for tempering, which is more difficult than in the case of excavators that are only plunged red hot into hard wax. But the pluggers I first heat sufficient to melt a small quantity of soap which prevents oxidizing and destroying the properties in the steel when subjected to so high a degree of heat as is necessary. The instrument is then heated to a very light *cherry red* and plunged into water, this will be a glass temper which is entirely too hard and must be reduced. First remove the oxide from the instrument with an emory stick so as to have a smooth and bright surface, that when the heat is applied I can see the different colors conducted down to the point. The flame is brought in contact about one inch from the point, and when the serrations are of a *straw color*, and the shank a *dark blue*, I plunge again in water, this will cause the serrations to be hard enough so as to retain their shape when brought in contact with gold and the hard tissues of the teeth, and will not break; the shank being a dark blue will support all the pressure that is necessary.

Sometimes you may find it difficult in keeping the points or serrations a straw color while the shank is of a dark blue, but if you place the points in a drop of water upon a cold hammer you can retain this color without any trouble. The instrument is now ready for polishing, which is done in the same manner the excavators are.

---

